# The Latest Surgical Triumph — the Transplantation of Eye from Rabbit to Man

**Published:** 1885-07

**Authors:** 


### THE LATEST SURGICAL TRIUMPH.

We copy the following from the *Journal of the American Medical Association*, of June 24: "TRANSPLANTATION OF AN EYE FROM A RABBIT TO A MAN. At the meeting of the Academie de Medecine of Paris, on May 28, M. Chibret reported that on May 4,1885,he had successfully transplanted an eye from a rabbit to a young girl, who had lost her left eye. The report, contained in the *Bulletin de l'Academie de Medecine*, does not say whether "successfully transplanted" means that the girl can see with the rabbit's eye or not; though one would infer that she can. It seems scarcely necessary to say, however, that such a result could not be hoped for, and that the operation must have been performed for cosmetic purposes only. Even in this case, the result should have stated whether or not the transplanted eye became controllable by the patient, and whether, if controllable, the movements are in accord with those of the other eye. If not, a glass eye would serve a better purpose. The report is characterized by that lack of detailed information which is too common in the published proceedings of the Paris Academy of Medicine.

"It would be still further interesting to know if the transplanted eye will grow in consonance with the other. We hope that the subsequent history of this very interesting case will be more fully reported."

We extracted the above with the intention of commenting upon it; but, on reflection, we feel we are not equal to the emergency, and, therefore, submit it, on its merit, to our readers. We will only say that our ambitious ophthalmologists, Salm, Chilton, Hall, Hodges and others will have to look to their laurels, and, if they do not wish to be eclipsed by their French brethren, they will have to be up and doing. They must report something in their TEXAS JOURNAL to beat it.

